# Identification of Guide-Intrinsic Determinants of Cas9 Specificity

**DOI:** 10.1089/crispr.2019.0009

**Published:** 2019-06-21

**Authors:** Nicholas C. Huston, Josh Tycko, Eric L. Tillotson, Christopher J. Wilson, Vic E. Myer, Hariharan Jayaram, Barrett E. Steinberg

**Affiliations:** Editas Medicine, Cambridge, Massaschusetts.

## Abstract

Considerable effort has been devoted to developing a comprehensive understanding of CRISPR nuclease specificity. *In silico* predictions and multiple genome-wide cellular and biochemical approaches have revealed a basic understanding of the Cas9 specificity profile. However, none of these approaches has delivered a model that allows accurate prediction of a CRISPR nuclease's ability to cleave a site based entirely on the sequence of the guide RNA (gRNA) and the target. We describe a library-based biochemical assay that directly reports the cleavage efficiency of a particular Cas9–guide complex by measuring both uncleaved and cleaved target molecules over a wide range of mismatched library members. We applied our assay using libraries of targets to evaluate the specificity of *Staphylococcus aureus* Cas9 under a variety of experimental conditions. Surprisingly, our data show an unexpectedly high variation in the random gRNA:target DNA mismatch tolerance when cleaving with different gRNAs, indicating guide-intrinsic mismatch permissiveness and challenging the assumption of universal specificity models. We use data generated by our assay to create the first off-target, guide-specific cleavage models. The barcoded libraries of targets approach is rapid, highly modular, and capable of generating protein- and guide-specific models, as well as illuminating the biophysics of Cas9 binding versus cutting. These models may be useful in identifying potential off-targets, and the gRNA-intrinsic nature of mismatch tolerance argues for coupling these specificity models with orthogonal methods for a more complete assessment of gRNA specificity.

## Introduction

RNA-guided DNA endonucleases (RGENs) such as Cas9 and Cas12a are highly efficient on-target cutting enzymes that have also been shown to exhibit off-target activity both biochemically and in cells. In therapeutic and other applications of RGENs, off-target genome cleavage presents a potential safety concern. Indeed, several studies have shown a guide RNA (gRNA):genomic DNA mismatch tolerance for Cas9 cleavage, highlighting the importance of understanding how the Cas9–guide ribonucleoprotein (RNP) mediates off-target cleavage.^1^ Several techniques do exist to probe off-target Cas9 cleavage in whole genomes, such as GUIDE-seq,^1^ Digenome-seq,^2^ BLISS,^3^ BLESS,^4^ CIRCLE-seq,^5^ and SITE-seq,^6^ but the sequence space of genomic substrates queried (e.g., from immortalized cell lines or healthy human donors) restricts analysis of off-target cleavage to genomic sites captured therein. Due to normal genetic variation,^7^ these empirical methods are insufficient to identify off-target cleavage events that may occur across populations. The analysis of off-targeting across species faces a similar limitation. Additionally, cell line–dependent factors such as chromatin state and cell-cycle status limit the subset of possible cleavage sites making off-target generalizations difficult to make.^8^ The small number of guide sequences and sequence constraints of sampled genomes tested to date prove insufficient to model the mismatch profile of CRISPR nucleases comprehensively. Understanding the trends governing Cas9 off-target cleavage requires data on disparate guides with large arrays of unbiased mismatched targets beyond those accessed through physically available whole-genome sequence space.

In order to avoid the constraints imposed by the sequences available in an experimental genome, researchers have instead used *in vitro* biochemical approaches, typically with synthetic libraries of DNA, to characterize the capability of *Streptococcus pyogenes* Cas9 to cleave mismatched DNA.^9,10^ These methods have established important principles of Cas9 activity such as the importance of the protospacer adjacent motif (PAM)-proximal seed region in governing binding and cleavage, while PAM-distal mismatches are better tolerated. However, existing models^11–13^ are largely universal: though built from a limited set of observations made of a limited set of guides, they are generalized to all complexed guides. Additionally, these methods typically rely on either enrichment^9^ or de-enrichment^10^ of cleaved targets. In these examples, only one cleavage state is quantitated. Without data for both cleaved and uncleaved molecules, an absolute efficiency metric in large libraries is unobtainable. This absolute efficiency metric is essential to compare off-target cleavage efficiencies accurately and to understand the dose–efficacy relationship of Cas9 adequately, a key requirement for developing therapeutic applications.

Alternative strategies include using catalytically inactive Cas9 (dCas9) binding as a proxy for off-target activity.^12,13^ However, the relationship between binding and cutting is complex,^14^ and the interaction of these states has not been evaluated for more than a few sequences. Despite our appreciation of the complexity, these approaches have not generated actionable quantitative models to predict safety or specificity.

We sought to design a biochemical assay to address these limitations by reporting the fractional cleavage of individual targets of a library composed of approximately 10^6^ unique members. This barcoded library of targets (BLT) approach is used to sample mismatched targets randomly and to report cleavage efficiency quantitatively. To achieve this, our method uses probe capture to amplify both cleaved and uncleaved molecules, enabling a highly quantitative determination of target-specific cleavage efficiencies. Synthetic library design allows for programmable diversity, such as robustly sampling arrays of mismatched targets, without being limited to a genomic sequence space. Moreover, the modularity of the process enables definable experimental conditions. We apply this method to generate off-target cleavage profiles of *Staphylococcus aureus* Cas9, a nuclease with few existing reports of off-target cleavage^15^ and a size (3.2 kb) well suited for delivery via adeno-associated virus (AAV) in therapeutic settings. Using BLT to quantify Cas9 cleavage efficiency allows for greater accuracy and control in measuring Cas9 cutting, in turn enabling improved modeling and understanding of the biophysical mechanisms underpinning Cas9 activity.

## Methods

### Library synthesis

Single stranded DNA (ssDNA) template was ordered through Integrated DNA Technologies (IDT), with 10% programmed degeneracy at each base of the wild-type (WT) target. A list of WT sgRNA and spacer sequences used in this study is available in Supplementary Table S4. At any given base position within a synthesized template library, 90% of template target contained the WT base, with the remaining 10% of templates containing any of the other three bases. Constant sequences 5′ of the target included an EcoRV cut site and a priming site for template library preparation. Constant sequences 3′ of the target included a PAM, a randomized unique molecular identifier (UMI) for target identification (UMI_T_), and a P7 priming site (Supplementary Table S1).

### Library bottlenecking

Template libraries received from IDT were brought to 100 μM in low TE (10 mM Tris-HCL, 0.1 mM EDTA, pH 8.0) and then serially diluted 1,000-fold in 10× increments. Concentrations of each dilution were determined with the Qubit ssDNA Assay Kit (Qubit Q10212) and diluted to 250,000 copies/μL. Libraries were polymerase chain reaction (PCR) amplified with approximately 62,500 template copies per reaction across eight technical replicates, or 500,000 templates total. All PCR reactions were prepared with 25 μL 2× NEBNext Ultra II DNA Library Prep Kit (NEB E7645S) and 0.5 μM of P7-Adaptor and Primer_T_ primers. Reactions were run at 95°C for 1 min, 27 cycles of 94°C for 30 s, 60°C for 30 s, and 72°C for 30 s, with a final extension at 72°C for 7 min. The PCR product was concentrated with the Zymo DNA Clean & Concentrator Kit (Zymo D4004) to facilitate downstream reaction preparation.

### RNP preparation

Component parts for two-part synthetics, including both the crRNA and tracrRNA, were ordered as RNA from IDT (Supplementary Table S2). gRNA annealing reactions were prepared with 7.5 μL of both 100 μM crRNA and 100 μM tracrRNA, 3 μL 10× annealing buffer (100 mM Tris-HCL, 500 mM NaCl, 10 mM EDTA), and 12 μL nuclease-free (NF) water. Reactions were annealed at 90°C for 5 min and a 2% ramp to 25°C. Final complexed gRNA concentration was 25 μM. *S. aureus* Cas9 was ordered from Aldevron with a 6× C-terminal His-tag and at a stock concentration of 50 μM. Protein was stored at −80°C until used.

All RNP was complexed using freshly prepared two-part synthetics. Complexation reactions were prepared with 4 μL *S. aureus* Cas9, 2 μL 10× H300 buffer (100 mM HEPES, 3 M NaCl, pH 7.5), 8.8 μL two-part synthetics, and 5.2 μL NF water. Reaction components were combined in the order listed. The complexation reaction was left to incubate at room temperate for 15 min and then immediately used in cutting assays. The final RNP concentration was 10 μM.

### Cutting reaction

The desired ratio of *S. aureus* Cas9 RNP:dsDNA template dictated the reaction set-up and was concentration dependent, ranging from 1:1 to 10:1. All cutting reactions were prepared to a final volume of 10 μL, with the constant addition of 1 μL 10× H300 and 2 μL 10 mM MgCl_2_. A typical cutting reaction contained final concentrations of 2.5 nM *S. aureus* Cas9 RNP and 0.5 nM dsDNA template for a final RNP:template of 5:1. For cutting reactions run in the presence of human gDNA, gDNA was present at a final concentration of 750 ng/μL. Reactions were run at 37°C for either 30 min or 16 h.

Following completion of the Cas9 cutting reaction, samples were immediately digested with EcoRV enzyme. Briefly, 10 μL 10× CutSmart (NEB), 5 μL EcoRV-HF (NEB), and 75 μL NF water were added to a 10 μL sample. EcoRV digest proceeded at 37°C for 30 min. The digested product was then concentrated with the Zymo DNA Clean & Concentrator Kit (Zymo D4004) and eluted in 10 μL of nuclease-free water.

### Probe preparation and ligation

Probes were ordered as ssDNA molecules from IDT (Supplementary Table S3). Constant sequences included a 5′ P5 Illumina adaptor, a nucleotide stagger ranging from 1 to 8 bp, two discrete 6 bp anchoring sequences, a fully randomized barcode for multiplexed sequencing reactions, a UMI probe (UMI_p_) to control for PCR bias and identify unique ligation events, and a constant 3′ anchoring sequence to facilitate probe amplification.

Probes were PCR amplified prior to use. All PCR reactions were prepared with 25 μL 2× NEBNext Ultra II DNA Library Prep Kit (NEB E7645S), 0.5 μM of both P5 adaptor and Primer_P_ primers, and 0.18 μL 100 μM probe. Reactions were run at 95°C for 1 min, 20 cycles of 94°C for 30 s, 60°C for 30 s, and 72°C for 30 s, with a final extension at 72°C for 7 min. The PCR product was concentrated with the Zymo DNA Clean & Concentrator Kit (Zymo D4004) and normalized to 30 ng/μL.

All ligation reactions were prepared using 5 μL of digested template library, 3 μL of 30 ng/μL probe, 1 μL 10× T4 Ligase buffer, and 1 μL high concentration T4 Ligase (NEB M0202M). Ligation reactions were performed at room temperature for 1 h.

### Library sequencing preparation and quality control

Following probe ligation, template libraries were PCR amplified for sequencing on the Illumina Miseq platform. All library amplification reactions were prepared using 25 μL 2× NEBNext Ultra II DNA Library Prep Kit (NEB E76455), 0.5 μM of both P5 adaptor and P7 adaptor primers, and 2 μL of ligated DNA template. All PCR reactions were prepared in triplicate and pooled post PCR for increased yield. Reactions were run at 95°C for 1 min, 14 cycles of 94°C for 30 s, 60°C for 30 s, and 72°C for 30 s, with a final extension at 72°C for 7 min. Pooled samples were visualized on 1% E-Gel Pre-Cast Agarose gels (Invitrogen) to verify successful amplification. To ensure equivalent read distribution between samples, relative band intensities were used to determine library pooling.

Once pooled, the library was again visualized on a 1% E-Gel Pre-Cast Agarose gels (Invitrogen). The region between 200 and 350 bp was excised. DNA was recovered using the Zymoclean™ Gel DNA Recovery Kit (Zymo D4001T) and eluted in 20 μL of nuclease-free water. The concentration of the final library was determined using the Qubit™ dsDNA HS assay kit (Invitrogen Q32851).

For Miseq loading, a 10 pM dilution library was prepared, including the addition of a 10% PhiX library spike-in to maintain cluster diversity. The Miseq Reagent Kit v2 was used for data collection, with 150 bp single reads used for data analysis.

All libraries shown were analyzed over at least three replicate reactions, and efficiency of each target was averaged.

### Data analysis

FASTQ files were parsed and demutiplexed according to probe barcode. Constant template and probe sequences, including probe UMI_p_, PAM, and template UMI_T_, were extracted. Mean read quality across these sequences, instead of the entire read, was determined. Any read with a mean read quality below Q20 was discarded.

After filtering on quality, reads were collapsed into “observations,” or any read that shared the same UMI_T_, UMI_P_, cut/uncut status, and probe stagger length. Targets were filtered by requiring at least three unique observations of a given uncut template–UMI_T_ combination, as UMI_T_ was used to identify target sequences cleaved by Cas9. It should be noted that it is impossible to validate targets that experience 100% cleavage, thus excluding them from analysis, therefore, naïve libraries were spiked in to aid in target validation. Remaining observations were then grouped across samples by guide, PAM, and UMI_T_, and were used to calculate cut fraction.

### Individual template biochemical testing

Cleavage efficiency was measured by a dose–response of RNP against 2 nM DNA substrate for 1 h at 37°C. RNP was added and mixed with DNA substrate using a Biomek FX^p^ liquid handler. Activity was quenched by the addition of Proteinase K followed by RNase If (NEB). Fraction of cleaved substrate was measured using the Fragment Analyzer automated CE system (AATI). Analysis was performed with GraphPad Prism using a four-parameter dose-response curve.

### Guide-specific neural network modeling

We reformatted mismatches of the target to the guide as a 4 × 21 matrix, with 1s representing mismatches to A, C, G, or T (in rows 1–4, respectively) and 0s representing matched bases. This matrix was reformatted again to a single binary string (1 × 84). We then created a matrix consisting of each mismatch string in the BLT data. This matrix was inputted into the Matlab Neural Network toolbox with a vector of associated cutting efficiencies ${ \eta _i}$ as expected output. Ten neurons were used with the Lavenberg–Marquardt training algorithm. Models were trained on 70% of inputted data, with 15% reserved for validation and 15% reserved as untrained data. All possible genomic off-targets of up to six mismatches to a given target were input as binary strings and processed through the generated neural network model to create the distribution shown in Figure 4C.

### Ni-bead extraction

The Ni-NTA Magnetic Agarose Bead extraction kit (Qiagen) was used to analyze the template content and cut status of Cas9-bound dsDNA. The Cas9 cutting reactions were prepared with 33 nM dsDNA template and 330 nM *S. aureus* Cas9 RNP (10:1 RNP:dsDNA template). Cutting reactions were run for 30 min at 37°C.

To prevent precipitation of the reaction's high protein content upon bead addition, 1 μL of 0.5% Tween20 (Sigma Aldritch) was added immediately following completion of the *S. aureus* Cas9 cutting reaction, after which 10 μL of Ni-NTA magnetic bead suspension was added to the reaction. The reaction was mixed by pipette and incubated at room temperature for 1 h to allow sufficient time for protein-bead binding. The sample was placed on a magnetic rack for 1 min followed by aspiration of supernatant. Two 30 s washes with 1× H300 (10 mM HEPES, 300 mM NaCl, pH 7.5) were performed while on the magnetic rack. After removal from the magnetic rack, the pellet was re-suspended in 30 μL 1 M NaCl and eluted at room temperature for 5 min. Following elution, the sample was separated for 1 min on the magnetic rack. Supernatant was collected, concentrated with the Zymo DNA Clean & Concentrator Lit (Zymo D4004), and eluted in 10 μL NF water. Samples were immediately digested with EcoRV as described above. A fragment analyzer (AATI) was used to confirm there was no observable sample loss by analysis of nucleic acid sizing over two replicate reactions at initial flow through, in the wash fraction, in the final bound fraction, and in a reaction without bead binding as a positive control.

For a given target *i*:

\begin{align*}
 
{ { \rm { \eta } } _i } = \; \left( { { \frac { \Sigma { \rm { Cleave } } { { \rm { d } } _i } }  { \Sigma { \rm { Observation } } { { \rm { s } } _i } } } } \right) \left( { { \frac { \Sigma { \rm { Observation } } { { \rm { s } } _ { { \rm { on } } \_ { \rm { target } } } } }  { \Sigma { \rm { Cleave } } { { \rm { d } } _ { { \rm { on } } \_ { \rm { target } } } } } } } \right) \tag { 1 } 
\end{align*}

where η represents normalized cutting efficiency to the on-target cleavage efficiency. The factor $\left( { { \frac { \Sigma { \rm { Cleave } } { { \rm { d } } _i } }  { \Sigma { \rm { Observation } } { { \rm { s } } _i } } } } \right)$ represents the unnormalized cleavage efficiency of each library member.

\begin{align*}
GIM { P_i } = \; { \frac { \mathop \Sigma\nolimits_j^k \left( { { { { \mathop \eta \limits^ { - } } _i } } \left( N \right) } \right) }  { k - j } } \tag { 2 } 
\end{align*}

In this study, we maintained Guide-Intrinsic Mismatch Permissiveness (GIMP) score measurements, as measured for mismatches *N* between *j* = 1 and *k* = 4.

\begin{align*}
 
{ P_ { { \rm { cut } } , i } } = { \frac { \Sigma { \rm { Cleave } } { { \rm { d } } _i } \; { \rm { bound \;to \;Cas } } 9 }  { \Sigma { \rm { Molecule } } { { \rm { s } } _i } { \rm { \;bound \;to \;Cas } } 9 } } \tag { 3 } 
\end{align*}

## Results

### Barcoded libraries of targets report cleavage efficiencies at a wide range of mismatches

**FIG. 1.**
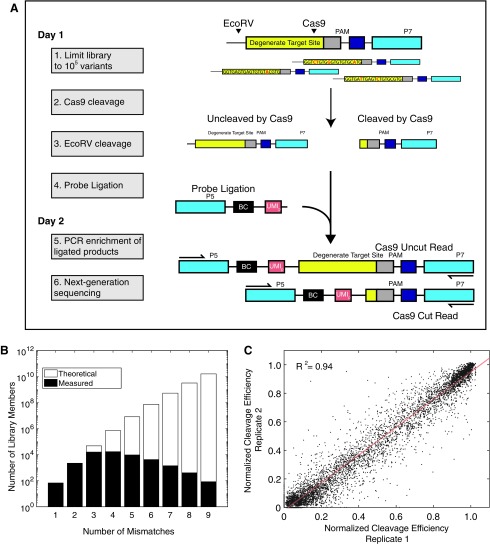
Barcoded library of targets (BLT) can measure Cas9 specificity at scale *in vitro*. **(A)** Schematic of barcoded library of targets experiment. Cleavage first by Cas9 and then by EcoRV allows measurement of cleavage efficiency by next-generation sequencing. Targets are identified by association with a unique molecular identifier (UMI). **(B)** Theoretical maximum library size and empirical distributions of mismatched targets analyzed in the data. **(C)** Cleavage efficiency measurements in BLT are highly reproducible across experiments.

A typical well-designed gRNA directed against a genomic locus has a single perfect match in the genome and few single or double genome-wide mismatches. However, once mismatches beyond three are considered, the spectrum of mismatched targets typically numbers in the hundreds to thousands. To evaluate large numbers of off-targets for a guide *in vitro*, we created a screening strategy using a barcoded library of targets (Fig. 1A). For each gRNA of interest, a template synthetic dsDNA library was generated with a constant PAM and a degenerate target site for that gRNA, with a 10% chance of random mismatch incorporation at each position relative to a given Cas9 guide (providing three median mutations per target; Fig. 1B). No indels were analyzed in our library. In order to quantitate uncleaved library members, we included an EcoRV site within the construct. After digestion with Cas9 RNP, the remaining uncut library members were cleaved with EcoRV. Cleavage events exposed a 5′ phosphate, enabling ligation of molecules to a dsDNA required for subsequent PCR amplification and sequencing. These ligated products were then amplified by PCR to create a sequencing library. The library was split between long (243 bp) reads and short (218 bp) reads, corresponding to ligation events at the EcoRV site or the Cas9 site, respectively. Counting the long and short reads provides a “snapshot” measure of the Cas9-cut fraction associated with a certain target sequence (equation 1). We find our measure of cutting efficiency to be highly reproducible across experiments (Fig. 1C), with *R*^2^ = 0.94 in our overall data set and reaching *R*^2^ = 0.98 by filtering for at least 50 reads analyzed per target.

**FIG. 2.**
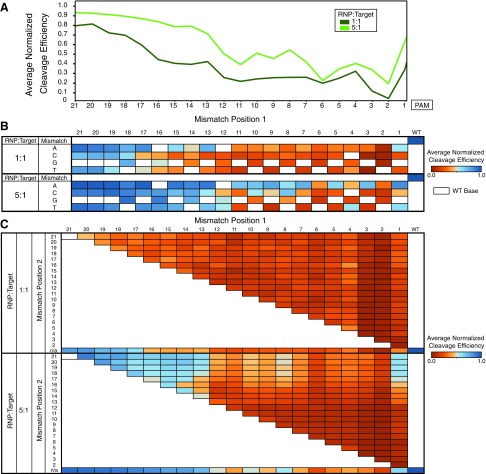
BLT can be used to evaluate the cutting efficiency of large libraries of mismatches. Reactions were run at either 5:1 RNP:target library or 1:1 RNP:target library for 30 min. Cleavage efficiency is normalized to the on-target cleavage efficiency, as noted in equation 1. **(A)** Cleavage efficiency is a function of mismatch position, shown on single mismatches of guide to target. Two ratios of VEGFA-targeting Cas9 RNP:target library are shown. Higher Cas9 dose increases efficiency but does not qualitatively alter the position dependence of mutations. Mutations within the seed region tend to cause lower cutting efficiency. **(B)** The efficiency of cutting at single mismatches is position and base dependent. White boxes indicate the wild-type (WT), unmutated base. **(C)** Doubly mismatched targets expand position-dependent trends in cutting efficiency, for example multiple protospacer adjacent motif (PAM)-distal mismatches still cut at high efficiency.

We applied the BLT approach to the previously studied promiscuous VEGF-A gRNA.^1,16^ Figure 2 shows the results of our quantitation for single mismatches (Fig. 2A and B) and double mismatches (Fig. 2C) on the gRNA. Our highly diverse libraries allow for evaluation of higher-order mismatches as well, showing intuitive reduction in Cas9 activity on average as mismatches accumulate (Supplementary Fig. S1B). We corroborate previous studies^9,10,17^ that highlight the importance of a seed region, evidenced here by drastically reduced cleavage efficiency in the presence of a single PAM-proximal mismatch. Increasingly PAM-distal mismatches show a commensurate reduction in the effect observed on cleavage efficiency (Fig. 2A). Our method also allows for assessment of the effect of mismatch type on Cas9 cleavage efficiency. Our results reliably show cleavage at select off-targets with a greater number of mismatches. However, we did not observe obvious or straightforward trends in sequence-dependence across guides. Unlike alternative screens which struggle to control library diversity, BLT methodologies can accurately measure efficiencies of progressively more mismatched targets. Combinations of mismatches often show complex behavior reflecting epistatic interactions, and make simple additive models of cleavage of higher-order mismatches difficult (Fig. 2C and Supplementary Fig. S1C). For example, two mutations within the first 10 bases of the target or the second 10 bases of the target reduce activity more than expected from the individual mutations, but one mutation each from the two halves of the guide tend not to interact epistatically (Supplementary Fig. S1C).

Due to the *in vitro* context of BLT, stoichiometry of target substrate and RNP complex and cleavage time can easily be adjusted. We see increased cutting as RNP dose increases, but similar relative profiles of cleavage by number of mismatches or position (Fig. 2). Likewise, increasing time of cutting from 30 min to 16 h shows an increase in efficiency but overall similar profiles. Previous research on the effects of Cas9 dose on off-target cutting^6^ have indicated that increasing dose and/or time reveals a more comprehensive cleavage profile in genomic sites. Our data are consistent with these results, showing greater propensity to cut a given sequence but largely maintaining sequence preference as nuclease dose increases.

In order to validate the BLT output further, we tested individual off-targets with “high,” “medium,” or “low” efficiencies of cutting, as reported by our library analysis (Supplementary Fig. S2). These off-targets and on-target sequences were exposed to increasing doses of Cas9 RNP. Individual *in vitro* tests verified that the rank order of maximum cutting efficiencies was similar to BLT library output. Biochemical testing confirmed that editing of each target plateaus in cleavage efficiency at increased doses with some dose-dependent variation, indicating an intrinsic editing ceiling for each guide–target pair.

### Randomized libraries indicate that mismatch tolerance is guide specific

**FIG. 3.**
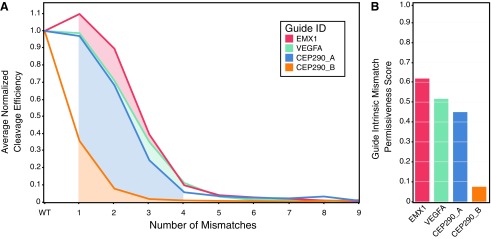
Mismatch permissiveness is guide intrinsic. The average mismatch tolerance of several guides on randomized mismatches varies widely when determined as a function of mismatch number. Reactions were run at 10:1 RNP:target library for 30 min. **(A)** A guide-intrinsic mismatch permissiveness (GIMP) score can be derived from this function by integrating over mismatch number (indicated by the shaded region). **(B)** The GIMP score varies widely among different guides. A high GIMP score indicates high intrinsic permissiveness of a guide to cleave indiscriminately. Low GIMP guides are intrinsically specific.

We next evaluated multiple guides using BLT (Fig. 3 and Supplementary Fig. S3). These seven guides included some guides designed to have lower mismatch coverage in the genome, as well as known promiscuous guides useful for studying off-target behavior. Because we performed sampling of randomized off-targets, we can evaluate the effects of guide choice upon off-target cleavage in a genome-independent experiment. Measuring the average decrease in cutting activity of seven guides at random mismatches to their respective targets shows that guides have distinct promiscuities (Fig. 3), implying a sequence-intrinsic (rather than purely position-specific) basis for Cas9–guide promiscuity. We can calculate a measure of this propensity by integrating the cleavage efficiency at random targets over increasing numbers of mismatches, generating a GIMP score (equation 2, Fig. 3, and Supplementary Fig. S3). Large GIMP scores indicate greater intrinsic promiscuity, while lower scores imply greater specificity. The GIMP metric allows for a large dynamic series, with GIMP scores ranging from ${ \rm{GIM}}{{ \rm{P}}_{{ \rm{CEP}}290 \_{ \rm{B }}}}$ = 0.082 to ${ \rm{GIM}}{{ \rm{P}}_{{ \rm{EMX}}1 - { \rm{sg}}1{ \rm{ }}}}$ = 0.62 found in our survey of guides. We hypothesize that this score represents the intrinsic tendency of a guide–Cas9 complex to cleave at any target, relative to the on-target efficiency. This score may reflect a combination of multiple factors, including potential ensembles of misfolded RNPs, PAM-proximal DNA–RNA interactions in the folded RNP complex, the propensity of the RNP to transition to its active conformation, and the R-loop complex unwinding the bound DNA with helicase-like activity. Guides not only varied in activity based on number of mismatches, but also by effect of mismatch position (Fig. 3 and Supplementary Fig. S3A), supporting sequence-dependent effects on mismatch cleavage. For example, mutations in the seed region of VEGFA and EMX1-sg1 guides show less impact than seed mutations of CEP290 guides (Supplementary Fig. S3). Individual biochemical testing of off-targets using EMX1-sg1 further verified library results (Supplementary Fig. S2B). Rank ordering by GIMP scores held constant at differing doses of Cas9 RNP (Supplementary Fig. S3D) and was not correlated with on-target cutting efficiency (Supplementary Fig. S3C). This result ensures that GIMP scoring is not an artifact of differential cutting efficiency. Unsurprisingly,^18^ the lowest GIMP score resulted from a 19mer guide (CEP290_B), indicating a length-dependent contribution to specificity.

**FIG. 4.**
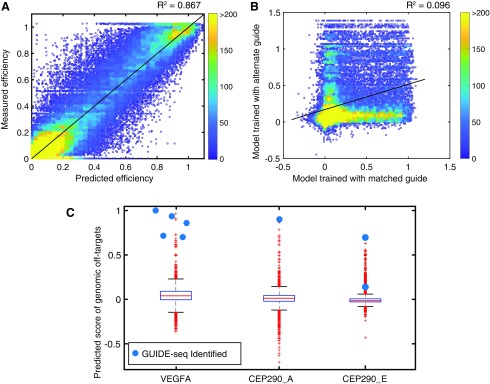
Guide-specific modeling predicts cleavage of off-target libraries *in vitro*. Neural network models were trained on data collected on reactions run for 30 min with 10:1 RNP:target library. **(A)** Predicted cutting efficiency versus measured efficiency of libraries of off-targets. Color indicates density of points. **(B)** Neural network models are highly guide specific. We applied either a prediction from a neural network model generated from a CEP290_C library or a prediction from a model generated from a VEGFA library, and applied these predictions to VEGFA off-targets. The results from each model are graphed. **(C)** Box plot of predicted efficiencies on genomic off-targets of *Staphylococcus aureus* complexed to VEGFA, CEP290_A, or CEP290_E (up to six mismatches). Sites identified by GUIDE-seq are indicated by larger blue dots.^17^

Typical approaches to predict Cas9 off-target cleavage incorporate a universal scoring function^19^ combined with known human genome sequence. However, the GIMP score adds an important guide sequence-specific consideration into the Cas9 RNP design. EMX1 and VEGF guides have both high frequencies of similar genomic sequences and high GIMP scores, suggesting they have a higher intrinsic propensity to cleave at a larger suite of off-target sites. Choosing a guide with a low GIMP score and low numbers of similar genomic sequences may decrease the overall risk of off-target cleavage.

### Guide-specific modeling

Because we observed guide-intrinsic promiscuity, we strove to create models for off-target cleavage, which were tailored to specific guide–Cas9 RNP complexes. BLT analysis with randomized targets provides rich data sets for quickly constructing models for a specific guide–protein complex. Our methods accurately captured targets with high numbers of mismatches, allowing us to avoid over-reliance on the effects of single mismatches, which are not usually additive (Supplementary Fig. S1C). We applied a neural network training algorithm to generate six unique guide-specific models (Fig. 4A). Training used mismatch types and positions as input, with normalized cutting efficiency ${{ \rm{ \eta }}_i}$ as output. We used the trained models to predict cleavage efficiency. Neural network training allows for high predictive power on untrained data. For example, using BLT randomized off-targets of the VEGFA RNP, neural network modeling explained *R*^2^ = 0.87 of the data (Fig. 4A). Models that performed best when trained on data from higher doses of Cas9, as these experiments produced a more dynamic range of cutting efficiencies, but models were also insensitive to changes in dose (Supplementary Fig. S4A) or time (Supplementary Fig. S4B). As predicted, models were highly tailored to specific guides and showed very little correlation when applied to data sets from other guide complexes (*R*^2^ = 0.096 when analyzing VEGFA data with the CEP290_C model; Fig. 4B). Distributions of predicted scores on randomized off-targets tended to be lower for guides with lower GIMP scores (Supplementary Fig. S5A). As with the GIMP score, modeling shows highly guide-specific mismatch tolerability. A previous model exists for *S. aureus*, which do not correlate well with our guide-specific models (*R*^2^ < 0.1), likely because this model is universal to all guides and is not focused on modeling multiple mismatches.^20^ Our analysis implies that for complex off-targets, RNP-specific models offer improved prediction of nuclease cleavage activity.

We then used our guide-specific model to score putative genomic off-targets with up to six mismatches in the reference human genome (Fig. 4C and Supplementary Fig. S5B). These scores were examined for GUIDE-seq identified off-targets for *S. aureus* Cas9 from a highly promiscuous (high GIMP score) VEGFA guide, the CEP290_E guide, and a more specific (low GIMP score) CEP290_A guide (Fig. 4C).^16^ The six sequences with multiple GUIDE-seq-identified reads all scored within the top 0.5% (*n* = 39/8,564) of possible off-targets in the genome (*n* ≤ 6 mismatches) for the VEGFA guide. The cellular validation of high BLT scoring guides with GUIDE-seq is very encouraging and underscores the utility of adding these sites to panels for targeted off-target sequencing under relevant treatment conditions. Other biochemical techniques, such as SITE-seq and Digenome, often show extensive cleavage that does not replicate in cells.^2,6^ Cellular reproduction of all off-target cleavage may require the perfect combination of conditions (cell type, gRNA format, etc.) to be validated by targeted sequencing. Importantly, the single identified GUIDE-seq off target for CEP290_A scored highest in predicted cleavage efficiency (*n* = 1/3,954). As a control, analysis of these off-targets with neural network models created from other guides did not typically show strong scoring, again indicating the ability to capture a nuclease-guide-intrinsic model with high fidelity.

Given this fidelity, improved modeling has clear use in identifying off-targets that may not be identified using GUIDE-seq or other methods to test via targeted next-generation sequencing. Previous studies attempting to assay the specificity of guides with *in vivo* systems have relied on “naïve” *in silico* models which detected no off-targets at the limited number of sites sequenced.^21,22^ However, *bona fide* cellular off-targets may contain multiple mismatches that either would be poorly modeled by existing scoring schemes^19^ or may yield lists that are too large to screen practically without any ranking criteria. Alternatively, BLT-derived guide-intrinsic models can yield a meaningful list of off-targets to assess safety in these model systems. For example, out of all 688 triply mismatched targets in the human reference genome, we identified two VEGFA off-targets, each with three mismatches as likely to cut. Targeted sequencing can be biased toward these highly scored sites, demonstrating that rank ordering of an extensive *in silico* list allows for greater focus on high-risk targets.

Additionally, because modeling is genome independent, our method should be able to predict high-risk off-targets across known genomes, chromatin states, or genomic variants. For example, 428 off-targets were scored in the top 5% of predicted VEGFA sites in the human genome. When the model is applied to the mouse genome, the top 5% of sites include 6,082 off-targets (with 82 common to both; Supplementary Fig. S5C and D). In contrast, screening all possible off-targets with up to six mismatches unique to the mouse would require analyzing 124,786 sites. Rapidly predicting and ranking unintended cleavage events when transferring nucleases between organisms will aid in better understanding the specificity behavior of Cas9 in model organisms *in vivo*, a prerequisite for clinical development of gene-editing therapeutics. Accurate and robust prediction metrics that can be applied across genomes are thus best enabled by guide-specific models.

### Cleavage efficiencies of targets bound to Cas9 quantitate cutting probabilities upon RNP complexing

Because BLT is highly amenable to variable experimental conditions *in vitro*, we thought to deploy our method to distinguish and control factors contributing to Cas9 cleavage from those contributing to Cas9 binding. The relationship between binding and cutting has been queried using FRET methodologies,^23,24^ but the efficiency of cutting once Cas9 has bound its target has not been quantified in detail. A detailed understanding of this relationship would advance understanding of Cas9 search activity and cleavage decisions, including at off-targets in the genome. For example, we see plateaus in cleavage efficiencies of on- and off-targets in Supplementary Figure S2. Understanding the ability of Cas9 to bind without cutting a target informs why these plateaus may result and may inform gRNA-intrinsic editing limitations in cells.

**FIG. 5.**
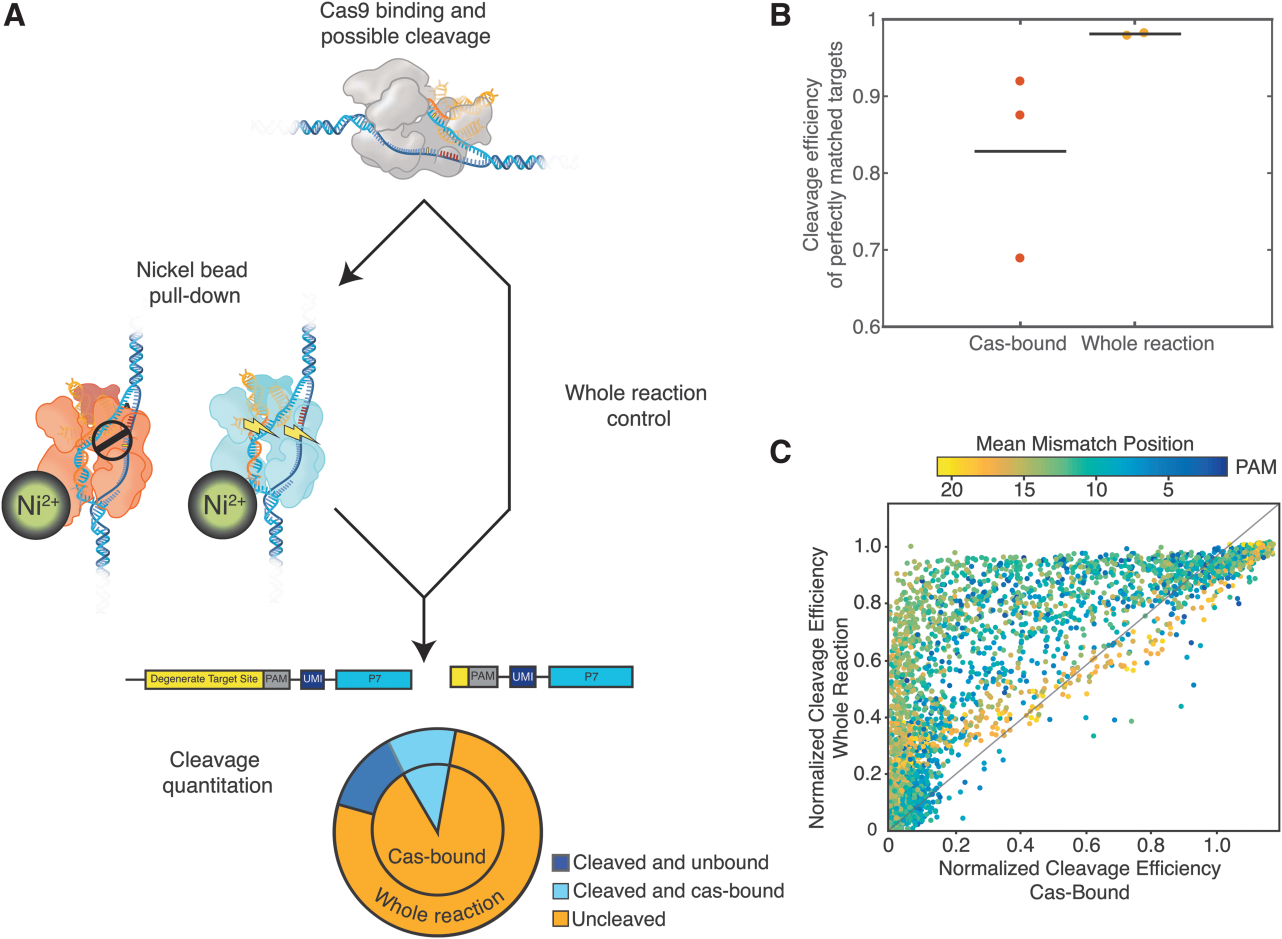
Cleavage efficiency of DNA once Cas9 is bound implies an intrinsic probability of cleavage success, P_cut_. **(A)** Schematic of measuring P_cut_ with BLT. Cas9 molecules are pulled down via nickel beads, and the cutting efficiency at bound targets is compared to a whole reaction control (including Cas-bound and unbound targets). **(B)** Captured Cas9-bound perfectly matched DNA contains a population of both cleaved and uncleaved target molecules. Dots represent average cleavage fraction of individual BLT experiments, while the line represents the mean fraction of cleaved on-target molecules. Reactions were run at 10:1 RNP:target library for 30 min. **(C)** Normalized cleavage efficiencies of a whole *in vitro* reaction and Cas9-bound library of off-targets. Color represents mean mismatch distance from the PAM. Many combinations of PAM-distal mutations fall along the line *y = x*, noted in black, which indicates no significant difference in cutting efficiency in the whole reaction versus Cas9-bound targets and greater reliance of overall efficiency on P_cut_ rather than binding efficiency (see also Supplementary Figure S6C).

Using a bead pull-down followed by BLT analysis on both the captured fraction of targets bound to Cas9 and a whole reaction control (containing both bound targets and free targets in reaction), we can measure the probability of Cas9 cleavage after binding (P_cut_; equation 3 and Fig. 5A). P_cut_ values approaching 1 suggest Cas9 cleaves successfully once bound, while P_cut_ values approaching 0 suggest cleavage does not occur once bound. We interpret this metric as a “snapshot” of overall bound Cas9 cleavage efficiency at the time of measurement. We confirmed that no loss of cleaved fragments that might bias our P_cut_ quantification occurred during wash or binding steps via capillary electrophoresis.

Figure 5B demonstrates that 1.9% of the on-target population in the whole reaction is uncleaved by the VEGFA RNP (*n* = 7,822/419,201 observations total), but in the Cas9-bound fraction, 17.7% of on-targets remained uncleaved (*n* = 62,587/353,065 observations total). Therefore, P_cut,VEGFA_ = 0.823 for the on-target molecule. P_cut_ values may also be guide specific, as testing on guide CEP290_B, a 19mer with a very low GIMP score, revealed 73% of on-target molecules in the whole population remained uncleaved (*n* = 162,692/221,824), while 99.8% of on-target molecules remained uncleaved when Cas-bound (*n* = 337,626/336,904; P_cut,CEP290_B_ = 0.002). Obtaining data on P_cut_ and GIMP scores across additional guides could potentially reveal stronger correlations between these metrics.

The BLT method extends P_cut_ analysis to off-targets, shown in Figure 5C. The large majority of mismatched targets show decreased cutting in Cas9-bound libraries compared to the whole reaction control (Supplementary Fig. S6A). This is consistent with multiple studies showing that not all bound off-targets are cleaved by their cognate RNP.^8^ Unlike these previous methods, this approach allows us to examine the position and mismatch base dependence of P_cut._ We show that P_cut_ has a strong position dependence (Fig. 5C and Supplementary Fig. S6B), with populations of Cas-bound targets that contain several PAM-distal mismatches showing greater parity with their whole-population counterparts when compared to targets with more PAM-proximal mismatches (Supplementary Fig. S6C). This result supports the structural inference that accumulating PAM-distal mismatches interferes with cleavage activation but does not interfere as strongly with binding.^25^

**FIG. 6.**
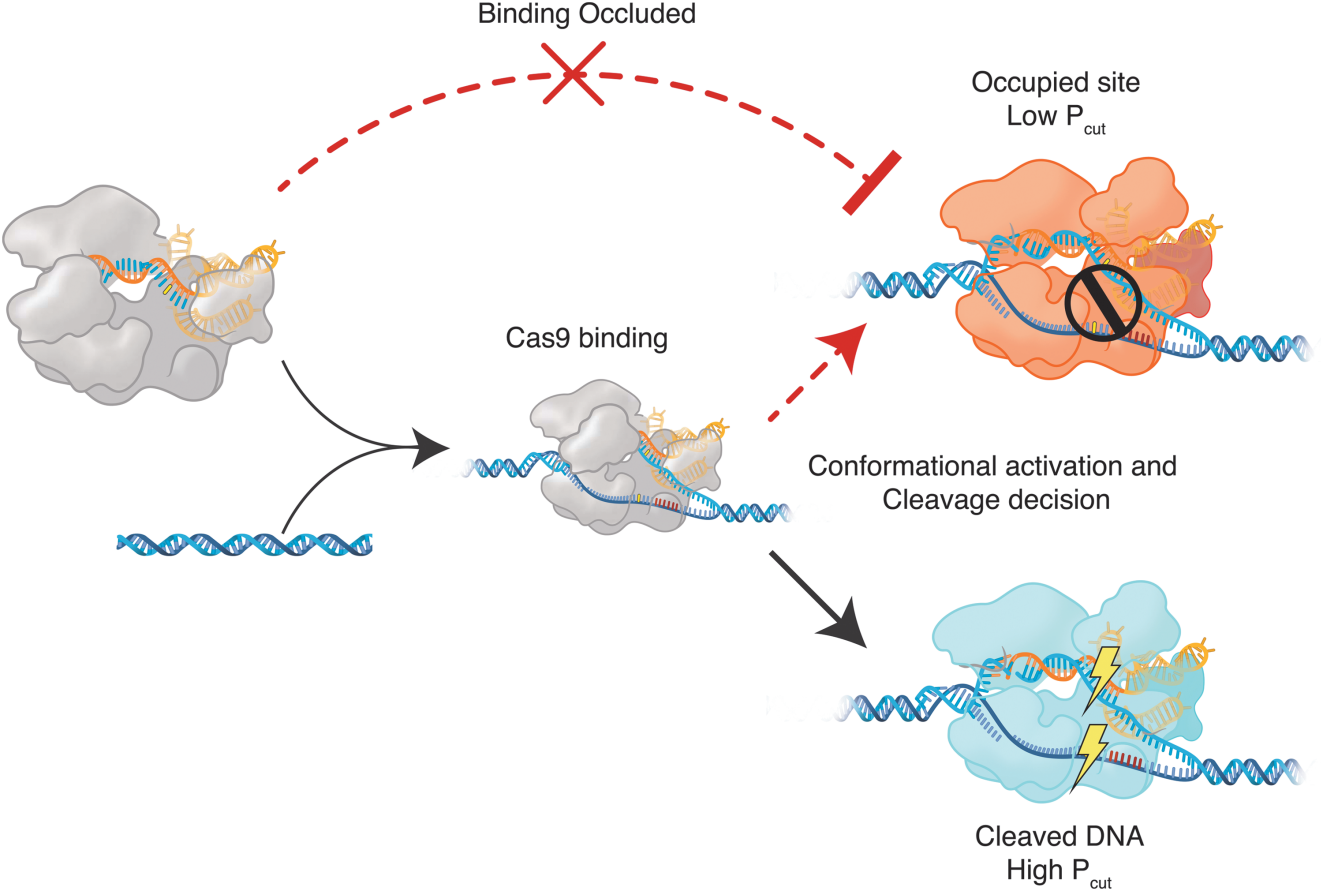
Cas9 molecules trapped in intermediate states may occlude target sites. In this model, a Cas9 RNP bound to substrate DNA is stuck in an inactive state (orange) and unable to cut a target successfully (i.e., low P_cut_). Cas9 with higher P_cut_ has greater chance to cleave successfully once bound (blue). This decision is made heterogeneously throughout a population of bound RNPs, wherein each target sequence has a distribution of cleavage states (see Fig. 5). Potentially active Cas9 RNPs are unable to access and cleave the occluded site. Occluding Cas9 is maintained on substrate DNA by slow dissociation rates.

The greater efficiency of cleavage in the whole reaction relative to the Cas-bound fraction implies that Cas9 RNPs can repeatedly sample targets, possibly by incomplete conformational activation during Cas9 interrogation. In this model, targets experiencing interrogation have an opportunity for Cas9 cleavage given by P_cut_ of that target–RNP complex. Throughout the experiment, certain targets with low P_cut_ but high overall efficiencies in bulk logically experience repeated opportunities for binding and cleavage over the time of the experiment, as noted in Supplementary Figure S6C. However, a population of Cas9 RNP may also remain in a bound, non-functional state on targets if its P_cut_ is low (Fig. 6). In this scenario, a failed cleavage attempt would trap a Cas9 molecule in a bound and inactive conformation, precluding additional cleavage attempts by free Cas9 (Fig. 6). Targets with similar efficiency measurements between the bulk reaction and the bound fraction are at highest risk for Cas9 occlusion (Supplementary Fig. S6C). Slow dissociation has been shown to maintain inactive Cas9 on a given site.^26^ Although *S. aureus* Cas9 has recently been shown to be a multiple turnover enzyme,^27^ it is believed that product release occurs after cleavage. Therefore, occlusion may still be responsible for inhibition of activity. This occlusion could explain plateaus in cleavage efficiency: a probabilistic fraction of bound Cas9 successfully cut, but an alternate population remains bound and prevents repeated cutting attempts, despite increasing reaction times or RNP dose. Our data illustrates that the relationship between binding and cleavage is distinctly target-specific and complex. We expect that our methods and data will complement existing data sets on Cas9 binding, such as via CHIP-seq.^12^

Cas9 conformational dynamics have been shown to act as a checkpoint on cleavage.^14,26^ Position-dependent effects on cutting given Cas9 binding corroborate models of RNA–DNA unwinding necessitating cleavage activation.^26^ BLT methods, however, allow large-scale evaluation and quantitation of the effects of mismatches and position on cleavage activation. In cells, on-target sites are often present only twice per cell, while Cas9 RNP is typically present at high relative stoichiometry to its targets, yet cleavage may not necessarily occur. Understanding the efficiencies of cleavage of various guides may inform an understanding of Cas9 biophysics and shape engineering efforts of RNA-guided nucleases.

## Conclusion

BLT offers improved quantitation of absolute cleavage efficiency *in vitro* at randomized mutations. Evaluating cleavage efficiencies quickly and efficiently allows us to investigate experimental factors such as the stoichiometry of RNP to target. Other complementary *in vitro* methods^6^ have determined high overlap between *in vitro* testing and cleavage in cellular targets. Due to the nature of our library, randomly accessing genomic sequence is relatively rare. Instead, we evaluate an unbiased library. Unbiased sampling enables systematic testing of Cas9–guide specificity at scale without being limited to genomic sequences. This analysis allows for the creation of highly guide- and protein- specific cleavage models. These models can be applied across genomes or to genomic variants and help to rank potential off-targets for further testing. Our BLT methodology produced novel models for *S. aureus* off-target activity with a diverse set of guides.

Our results challenge the paradigm that Cas9 and its complex guide are completely modular and that generalized rules across all guides are obtainable without sacrificing fidelity. We used unbiased target libraries to find that guide choice instead influences off-target propensity in a guide-intrinsic manner in our survey of seven guides. Cleavage efficiencies can then be used to calculate relative promiscuity of each enzyme–guide complex. We further show that Cas9 cleavage has a complex but quantifiable relationship to binding with both on-target and mismatched sequences. Measuring the probability of nuclease cleavage given Cas9 binding expands the biophysical understanding of Cas9 dynamics as well as potentially explaining ceilings in cleavage and editing. The combination of these analyses systematically binding/cleaving specificity for various RNPs.

The BLT approach is easily adaptable to nuclease orthologs and various experimental conditions. Relative promiscuity of nucleases can easily and quickly be evaluated with this approach, as the effect of guide design among orthologs remains to be investigated systematically. Additionally, BLT can readily be applied to synthesized arrays of designed sequences rather than randomized libraries. By evaluating quantitative activity data, BLT provides a controlled characterization of nuclease editing ability among a vast array of target sites. Understanding and modeling the unique effects of each gRNA–protein complex greatly enhances predictive ability and comprehension of gene editing outcomes.

Finally, the clear guide-intrinsic properties described herein further support the assertion that purely *in silico* approaches are currently not sufficient to identify all likely candidate sites in the human genome for interrogation with targeted sequencing methods. We routinely saw highly matched sites based on sequence alone ignored, while more mismatched sites were cleaved with high efficiency. These observations are consistent with previous studies performed in cells.^1,28^ Ranking an extensive *in silico* list of off-targets with an informed model allows for targeted analysis of cleavage-prone sites. BLT allows for in-depth analysis as well as modeling of particular Cas9–gRNA complexes. Overall, our BLT-based model enhances *in silico* prediction as an orthogonal and complementary approach to techniques such as GUIDE-seq and Digenome in evaluating gRNA specificity.

## Supplementary Material

Supplemental data

Supplemental data

Supplemental data

Supplemental data

Supplemental data

Supplemental data

Supplemental data

Supplemental data

Supplemental data

Supplemental data

## Supplementary Material

Supplementary Table S4

Supplementary Equation
