# Supplementary material for: Identification of Guide-Intrinsic Determinants of Cas9 Specificity
Source: CRISPR J. 2019 Jun 21;2(3):172–85. doi: 10.1089/crispr.2019.0009 (PMC6694761; doi:10.1089/crispr.2019.0009)
Supplement: Supplemental data [file Supp_Table4.docx]

**Table S4**. Spacers and target sites used in this study.

| **Guide** | **RNA Spacer** | **Target Site (23-mer)** | **PAM** |
| --- | --- | --- | --- |
| EMX1_sg1 | GGCCUCCCCAAAGCCUGGCCA | CAGGCCTCCCCAAAGCCTGGCCA | GGGAGT |
| VEGFA | GGGUGAGUGAGUGUGUGCGUG | GTGGGTGAGTGAGTGTGTGCGTG | GGGAGT |
| CEP290_A | GAGAAAGGGAUGGGCACUUA | TAAGAGAAAGGGATGGGCACTTA | ATGAGT |
| CEP290_B | GAGUAUCUCCUGUUUGGCA | TGTTGAGTATCTCCTGTTTGGCA | CAGAGT |
| CEP290_C | GAAUAGUUUGUUCUGGGUAC | GGAGAATAGTTTGTTCTGGGTAC | AGGGGT |
| CEP290_D | GAUGCAGAACUAGUGUAGAC | TAAGATGCAGAACTAGTGTAGAC | AGGAGT |
| CEP290_E | GUCACAUGGGAGUCACAGGG | TAAGTCACATGGGAGTCACAGGG | TAGGAT |
