# Supplementary material for: Identification of Guide-Intrinsic Determinants of Cas9 Specificity
Source: CRISPR J. 2019 Jun 21;2(3):172–85. doi: 10.1089/crispr.2019.0009 (PMC6694761; doi:10.1089/crispr.2019.0009)
Supplement: Supplemental data [file Supp_Table1.docx]

**Table S1**. Template sequences used in all experiments

| **Template Sequences** | **Sequence** |
| --- | --- |
| Upstream (Primer_T_, EcoRV) | GCCTGGACTCAACCGGACCCGGGGATATCTG |
| Target Site (23-mer + endogenous PAM) | Guide specific |
| Downstream (UMI_T_, P7-Adaptor) | GCNNNNNNNNNNNNTGACAGATCGGAAGAGCACACGTCTG AACTCCAGTCACGCTGCTATCTCGTATGCCGTCTTCTGCTTG |
