# Supplementary material for: Identification of Guide-Intrinsic Determinants of Cas9 Specificity
Source: CRISPR J. 2019 Jun 21;2(3):172–85. doi: 10.1089/crispr.2019.0009 (PMC6694761; doi:10.1089/crispr.2019.0009)
Supplement: Supplemental data [file Supp_Table2.docx]

**Table S2**. Guide sequence used in all experiments.

| **Two-Part synthetic guides** | **Sequence** |
| --- | --- |
| Direct Repeat | RNA Spacer + GUUUUAGUACUCUGUAAUUUU |
| Scaffold | AAAAUUACAGAAUCUACUAAAACAAGGCAAAAU GCCGUGUUUAUCUCGUCAACUUGUUGGCGAGAUUUU |
