# Supplementary material for: Identification of Guide-Intrinsic Determinants of Cas9 Specificity
Source: CRISPR J. 2019 Jun 21;2(3):172–85. doi: 10.1089/crispr.2019.0009 (PMC6694761; doi:10.1089/crispr.2019.0009)
Supplement: Supplemental data [file Supp_Table3.docx]

**Table S3**. Probe sequences used in all experiments.

| **Probe No.** | **Start (P5-Adaptor)** | **Stagger** | **Anchor 1** | **Barcode** | **Anchor 2** | **UMI_P_** | **End (Primer_P_)** |
| --- | --- | --- | --- | --- | --- | --- | --- |
| 1 | AATGATACGGCGACCACCGAGATCTACACTCTTTCCCTACACGACGCTCTTCCGATCT | GCGGAAGC | CGATCT | CACATACGCACTACG | TACGAC | NNNNNN | TTACCGAAGATAGCAGCCTAGTGGAACC |
| 2 | AATGATACGGCGACCACCGAGATCTACACTCTTTCCCTACACGACGCTCTTCCGATCT | GCGGAAG | CGATCT | CCTATACCCGAATCT | TACGAC | NNNNNN | TTACCGAAGATAGCAGCCTAGTGGAACC |
| 3 | AATGATACGGCGACCACCGAGATCTACACTCTTTCCCTACACGACGCTCTTCCGATCT | GCGGAA | CGATCT | TATACAATTCGCAGC | TACGAC | NNNNNN | TTACCGAAGATAGCAGCCTAGTGGAACC |
| 4 | AATGATACGGCGACCACCGAGATCTACACTCTTTCCCTACACGACGCTCTTCCGATCT | GCGGA | CGATCT | CCGGAGTAGGTCCTC | TACGAC | NNNNNN | TTACCGAAGATAGCAGCCTAGTGGAACC |
| 5 | AATGATACGGCGACCACCGAGATCTACACTCTTTCCCTACACGACGCTCTTCCGATCT | GCGG | CGATCT | ATTGCAAGGGCCCTT | TACGAC | NNNNNN | TTACCGAAGATAGCAGCCTAGTGGAACC |
| 6 | AATGATACGGCGACCACCGAGATCTACACTCTTTCCCTACACGACGCTCTTCCGATCT | GCG | CGATCT | TCCCGTCGTCCACAA | TACGAC | NNNNNN | TTACCGAAGATAGCAGCCTAGTGGAACC |
| ­­­­­­­­­­7 | AATGATACGGCGACCACCGAGATCTACACTCTTTCCCTACACGACGCTCTTCCGATCT | GC | CGATCT | TGCGTGACAAGCTCT | TACGAC | NNNNNN | TTACCGAAGATAGCAGCCTAGTGGAACC |
| 8 | AATGATACGGCGACCACCGAGATCTACACTCTTTCCCTACACGACGCTCTTCCGATCT | G | CGATCT | GCGCAGTCTTTCTGC | TACGAC | NNNNNN | TTACCGAAGATAGCAGCCTAGTGGAACC |
