# Supplementary material for: Identification of Guide-Intrinsic Determinants of Cas9 Specificity
Source: CRISPR J. 2019 Jun 21;2(3):172–85. doi: 10.1089/crispr.2019.0009 (PMC6694761; doi:10.1089/crispr.2019.0009)
Supplement: Supplemental data [file Supp_Data-F1-EQ1.pdf]

Supplementary Data

Supplementary Equation

$$\epsilon_{ab} = \log \frac{\eta_{ab}}{(\eta_a)(\eta_b)}$$

where ϵ_{ab} represents pairwise epistasis between the cleavage efficiency of an off-target with two mutations a and b relative to the on-target.

Supplemental Data

BLT analysis for all experiments described (see separate tab-delimited files)

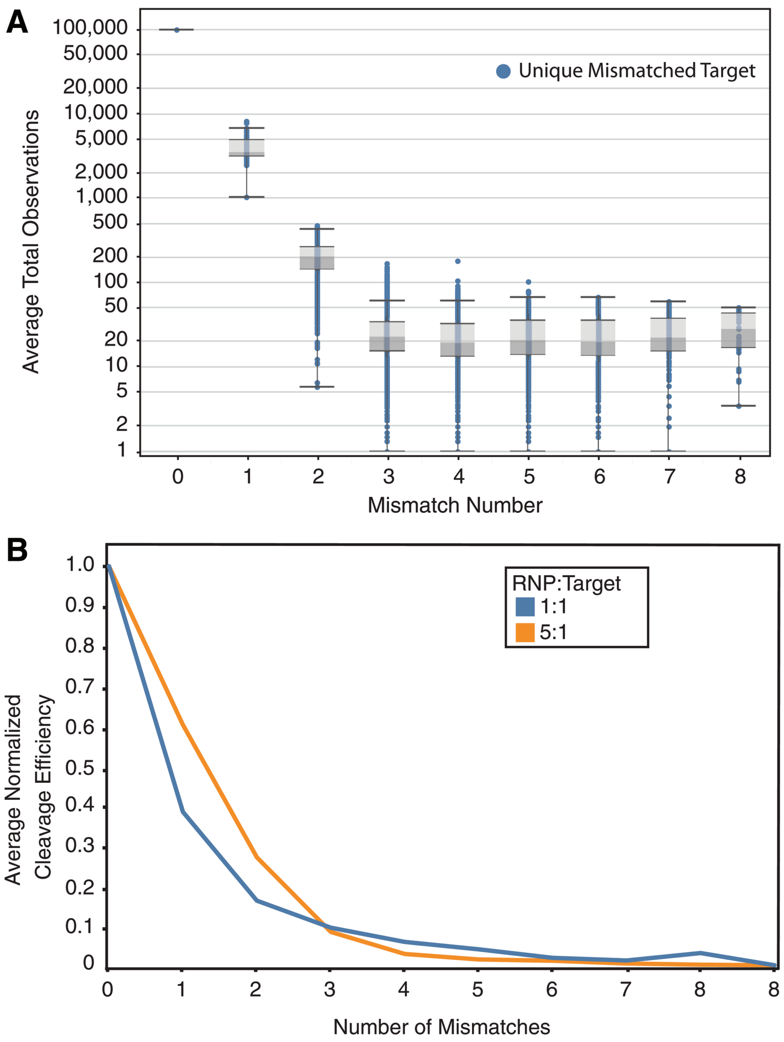

SUPPLEMENTARY FIG. S1. Barcoded libraries of targets methods robustly reveal trends in off-target cutting efficiency. **(A)** Depth of read coverage is maintained over library members with large numbers of mismatches. The box plot of average total observations for each unique mismatched target is graphed as a function of mismatch number. At high diversity targets, BLT maintains confident quantitation with reliably high numbers of observations. **(B)** Increasing numbers of mismatches and reduced dose tend to decrease cleavage efficiency. **(C)** Epistasis between off-target mutations is highly position dependent (see equation 4). Strong epistatic effects between two mutations relative to the VEGFA guide appear to be localized to either within bases 2–12 or within bases 12–21 of the target, indicated by blue clusters. However, one mutation from either region has little epistatic effect, indicated by the two yellow clusters. Reactions were run at either 5:1 RNP:target library or 1:1 RNP:target library for 30 min.

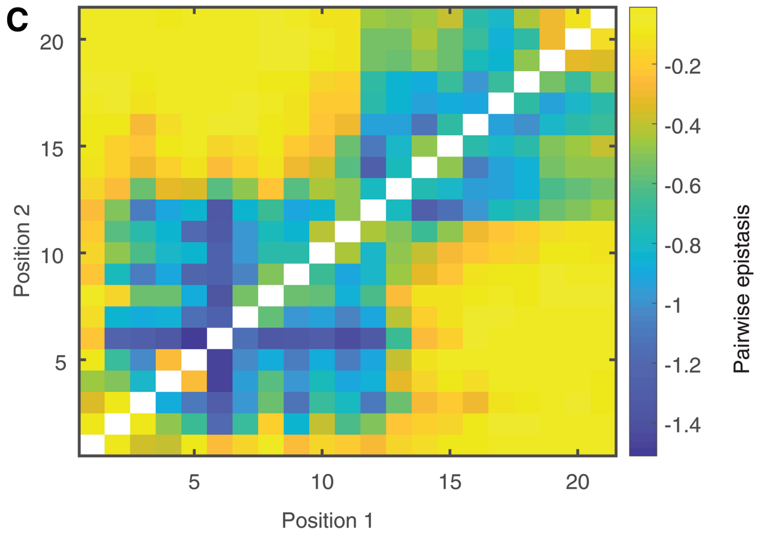

SUPPLEMENTARY FIG. S1. (Continued).
