# Supplementary material for: Identification of Guide-Intrinsic Determinants of Cas9 Specificity
Source: CRISPR J. 2019 Jun 21;2(3):172–85. doi: 10.1089/crispr.2019.0009 (PMC6694761; doi:10.1089/crispr.2019.0009)
Supplement: Supplemental data [file Supp_Fig2.pdf]

A

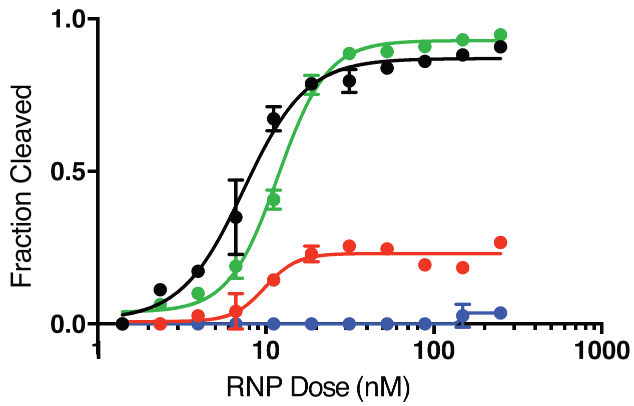

	Sequence	BLT cleavage efficiency	A_{max}
On-target	GGGTGAGTGAGTGTGTGCGTG	0.98	0.87
Off-target 1	GGGT**A**AGTGAGTGTG**A**GCGTG	0.97	0.93
Off-target 2	GGGTGA**T**TGA**T**TGTGTGCGTG	0.50	0.23
Off-target 3	GGGTGAGTGAGTGTG**G**GC**C**TG	0.05	0.04

B

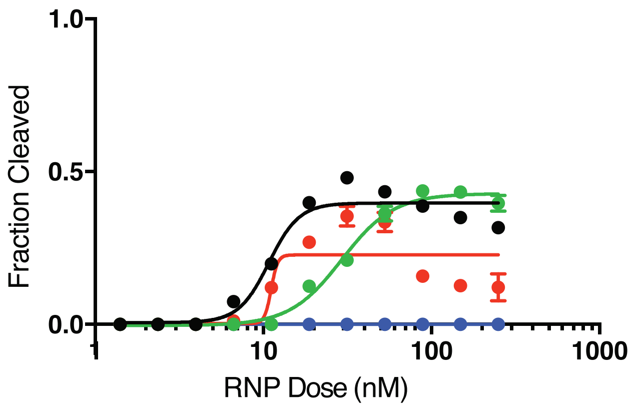

	Sequence	BLT cleavage efficiency	A_{max}
On-target	GGCCTCCCCAAAGCCTGGCCA	0.43	0.40
Off-target 1	GG**G**CTCCCCAAAGC**T**TGGCCA	0.69	0.43
Off-target 2	GGC**T**TCC**T**CAAAGCCTGGCCA	0.29	0.23
Off-target 3	GGCCT**G**C**G**CAAAGCCTGGCCA	0.08	0.00

SUPPLEMENTARY FIG. S2. Individual templates validate library results in biochemical cleavage assays. **(A)** Three representative doubly mismatched targets were subjected to increasing doses of VEGFA Cas9 RNP. Rank ordering of the cleavage efficiency in individual testing is consistent with BLT-measured results. A_{max} represents the maximum sigmoidal fitted cleavage efficiency. **(B)** Representative doubly mismatched targets from EMX1 off-target data were subjected to increasing doses of Cas9 RNP.
