# Supplementary material for: Identification of Guide-Intrinsic Determinants of Cas9 Specificity
Source: CRISPR J. 2019 Jun 21;2(3):172–85. doi: 10.1089/crispr.2019.0009 (PMC6694761; doi:10.1089/crispr.2019.0009)
Supplement: Supplemental data [file Supp_Fig3.pdf]

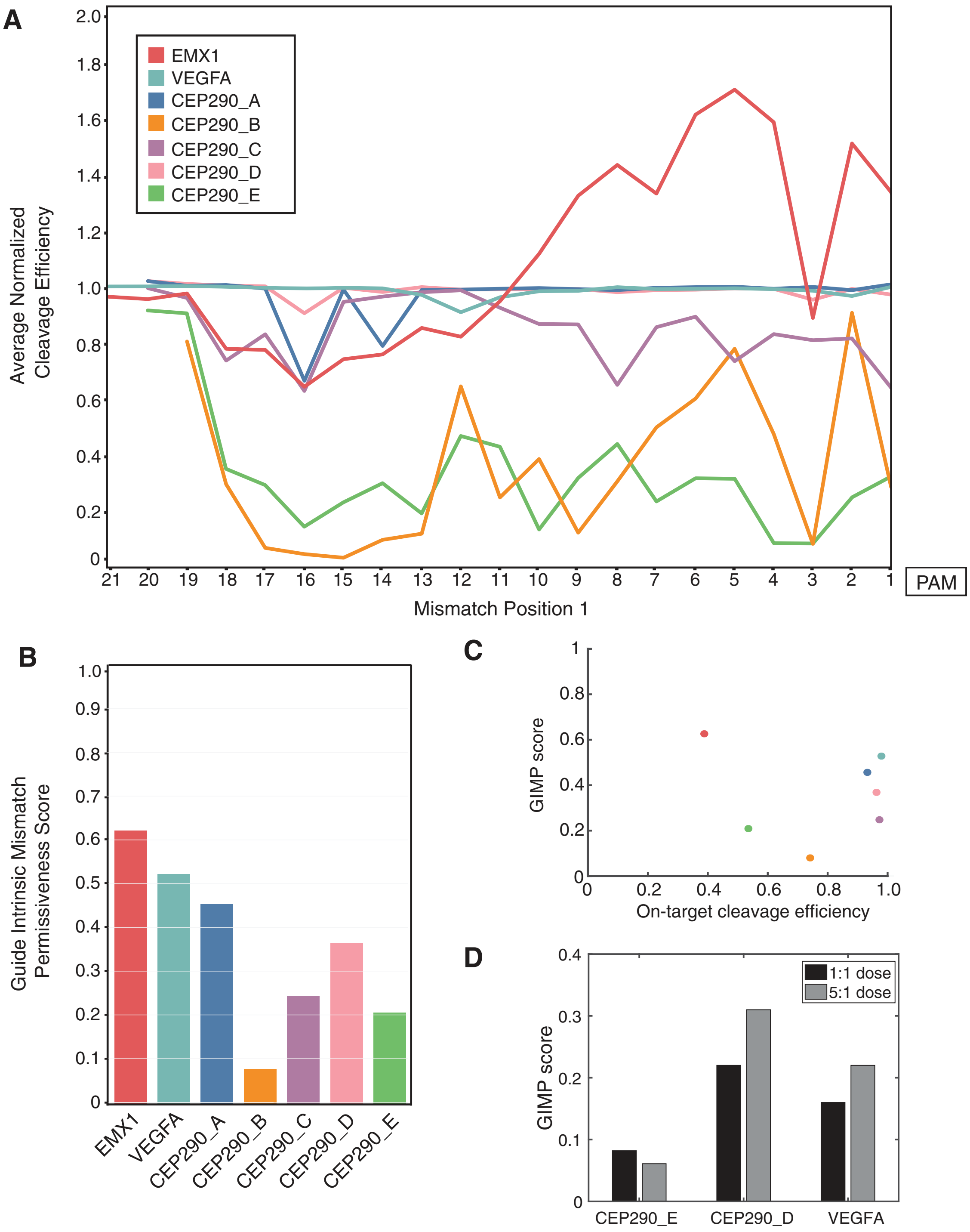

SUPPLEMENTARY FIG. S3. Mismatch tolerance is guide specific. Cleavage efficiency is normalized to the on-target cleavage efficiency, as noted in equation 1. **(A)** Guides have unique patterns of tolerance as a function of mismatch position. Average cleavage efficiency is graphed for all single mismatches across various targets. **(B)** Guides have unique GIMP scores when challenged with randomized off-targets. Reactions were run at 10:1 RNP:target library for 30 min. **(C)** Cleavage efficiency of a guide to its perfectly matched target does not correlate with GIMP score. GIMP score is graphed as a function of cleavage efficiency of the on-target ($R^2 = 0.01$). **(D)** GIMP score ranking is maintained with increasing RNP doses.
