# Supplementary material for: Identification of Guide-Intrinsic Determinants of Cas9 Specificity
Source: CRISPR J. 2019 Jun 21;2(3):172–85. doi: 10.1089/crispr.2019.0009 (PMC6694761; doi:10.1089/crispr.2019.0009)
Supplement: Supplemental data [file Supp_Fig4.pdf]

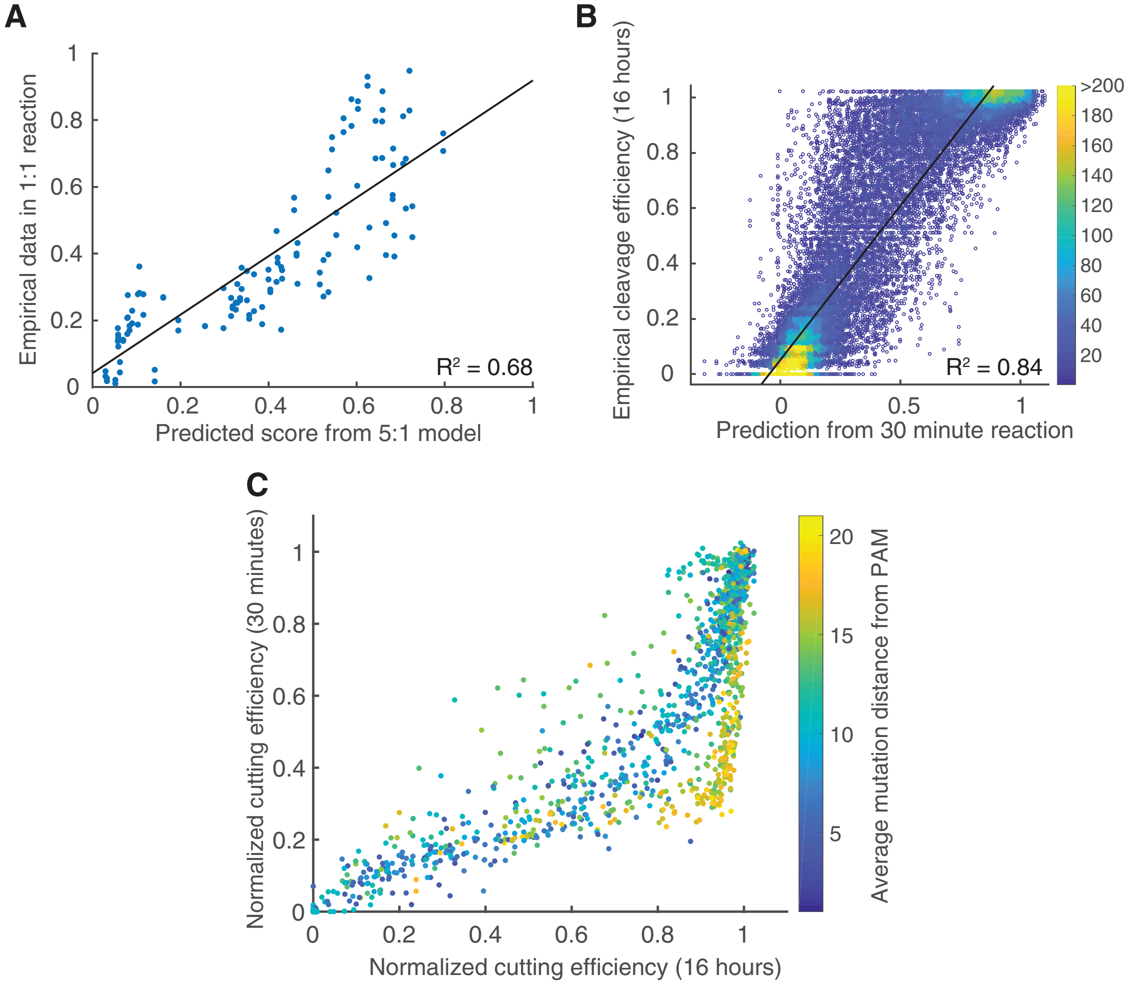

SUPPLEMENTARY FIG. S4. Neural network modeling is guide specific and robust to reaction conditions. (**A**) Modeling successfully predicts activity at lower RNP dose. A neural network model trained on data from the VEGFA RNP at 5:1 RNP:target library was applied to singly mismatched data from a reaction at 1:1 RNP:target library. The predicted score is graphed against the reported cleavage efficiency for each target, with the trend line indicated in black ($R^2 = 0.68$). Larger numbers of mismatches were not modeled due to lower efficiencies at lower doses reaching limits of detection due to poor sampling of the cleaved targets. (**B**) Modeling is insensitive to different time points. A neural network model trained on data from the VEGFA RNP with conditions of 10:1 RNP:target library for 30 min was applied to the library from the 16 h reaction. The predicted score is graphed against the reported cleavage efficiency for each target, with the trend line indicated in black ($R^2 = 0.84$). Color indicates density of points. **(C)** Increased cutting time increases cutting efficiency with slight PAM-distal bias using the VEGFA sgRNA. Reactions were run as in **(B)**. Normalized cleavage efficiency is graphed for targets that appear in both cleavage reactions. Points are colored by average mutation distance from the PAM. GIMP scores for the VEGFA RNP used in the 30 min and 16 hr cleavage reactions are 0.52 and 0.61, respectively.
