# Supplementary material for: Identification of Guide-Intrinsic Determinants of Cas9 Specificity
Source: CRISPR J. 2019 Jun 21;2(3):172–85. doi: 10.1089/crispr.2019.0009 (PMC6694761; doi:10.1089/crispr.2019.0009)
Supplement: Supplemental data [file Supp_Fig5.pdf]

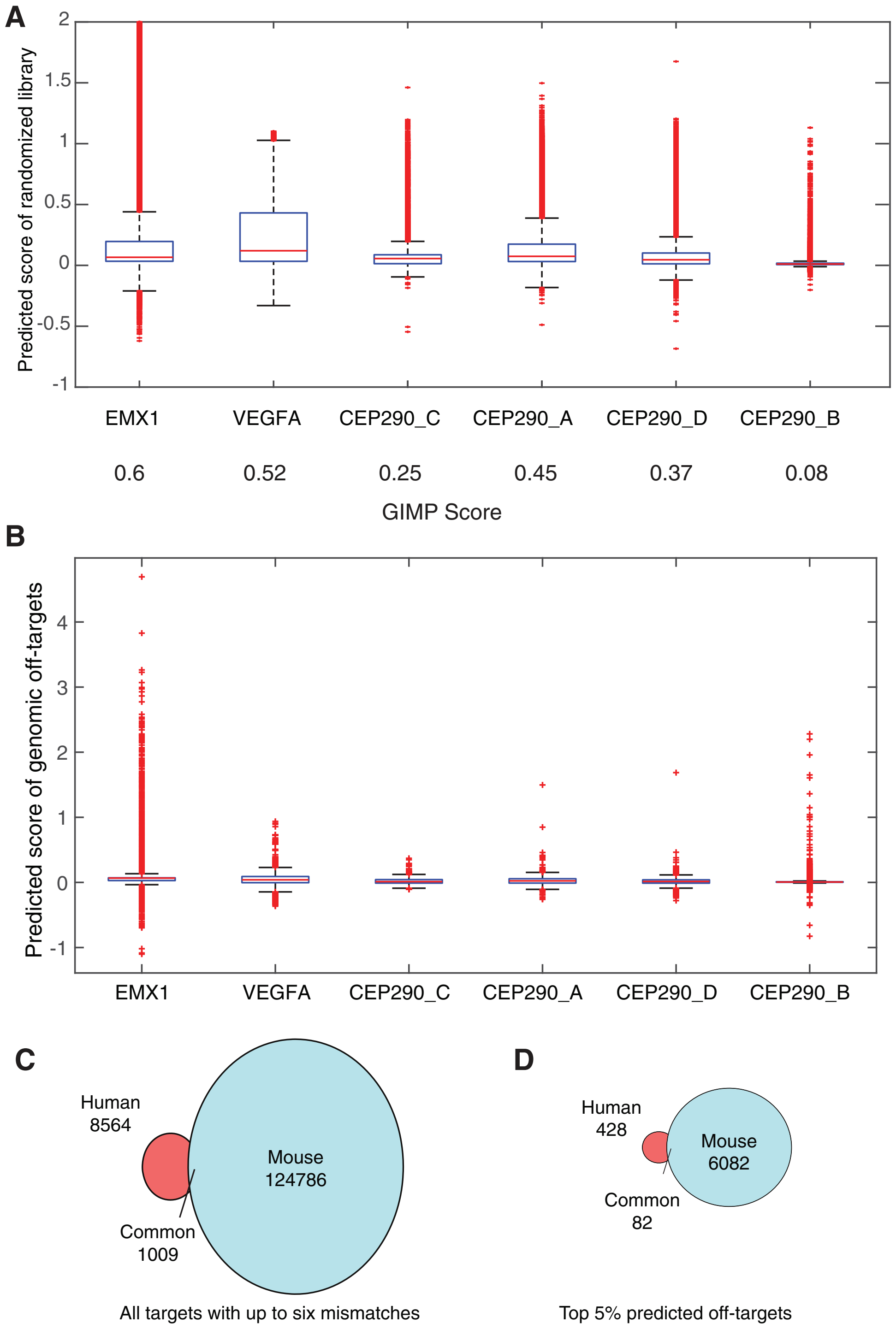

SUPPLEMENTARY FIG. S5. Guide-specific modeling predicts cleavage efficiencies in random libraries and in genomes. **(A)** Neural network training scores randomized target DNA across guide models. Box-plot distributions are shown for various guides surveyed. GIMP scores for each guide are listed below the *x*-axis. **(B)** Neural network training scores putative genomic off-target DNA across guide models. Box-plot distributions are shown for various guides surveyed on targets with up to six mismatches in the human genome. **(C)** Venn diagram of all off-targets with up to six mismatches in the human or mouse genome. **(D)** Venn diagram of only the top 5% of predicted off-targets in human or mouse genomes. **(C)** and **(D)** are not to scale with each other.
