# Supplementary material for: Identification of Guide-Intrinsic Determinants of Cas9 Specificity
Source: CRISPR J. 2019 Jun 21;2(3):172–85. doi: 10.1089/crispr.2019.0009 (PMC6694761; doi:10.1089/crispr.2019.0009)
Supplement: Supplemental data [file Supp_Fig6.pdf]

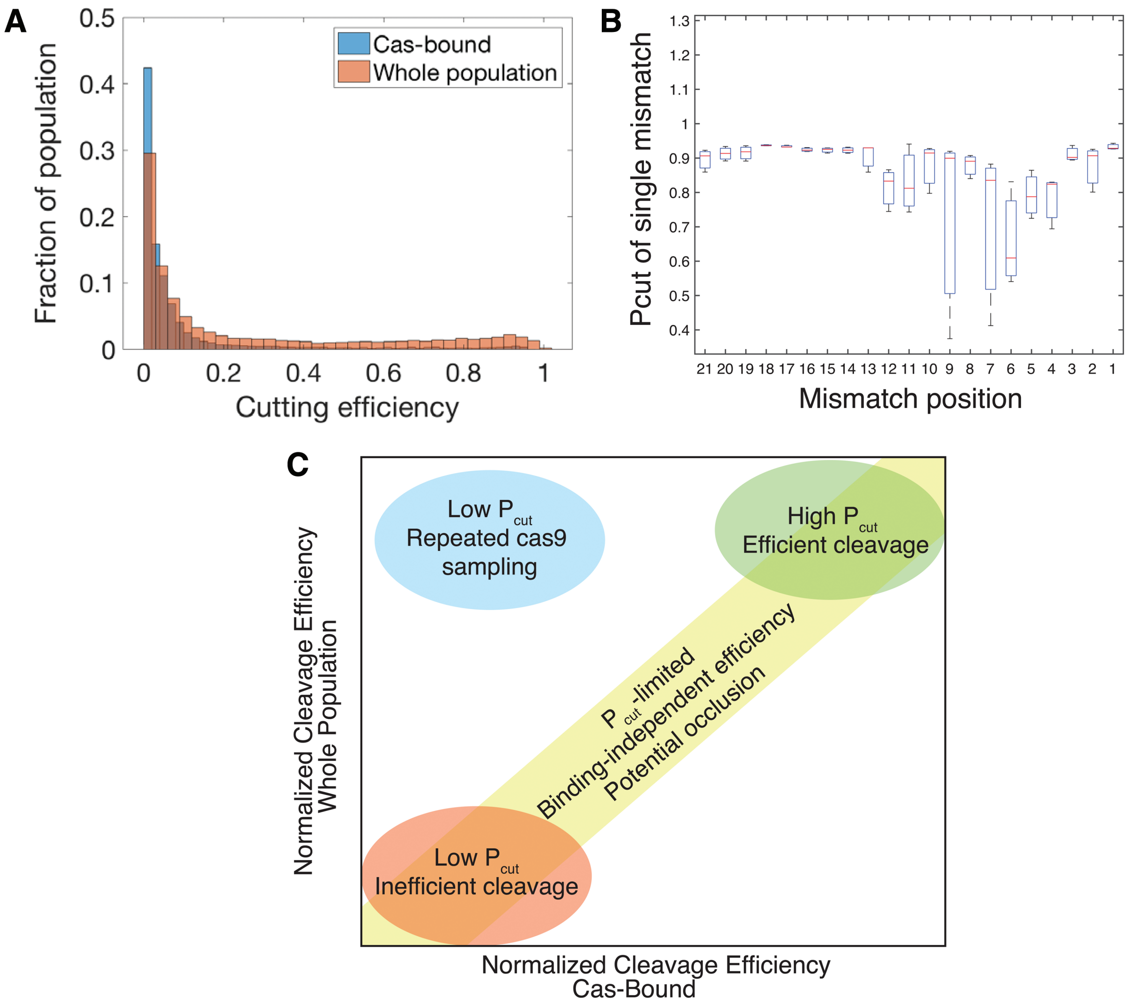

SUPPLEMENTARY FIG. S6. P_{cut} measurement informs biophysical explanations of cleavage outcomes. **(A)** Probability density functions of cleavage efficiency of Cas-bound and whole reaction controls differ. The histogram of Cas-bound cleavage efficiency is shown in blue, while the whole population is shown in orange. **(B)** Box plot of P_{cut} of single mismatches along the VEGFA target. Reactions were run at 10:1 RNP:target library for 30 min. P_{cut} demonstrates position dependence. **(C)** Measuring the efficiency of cutting in bound and unbound states informs limitations on Cas9 cleavage. Interpretation of Figure 5C.
